# Long-distance transport of L-ascorbic acid in potato

**DOI:** 10.1186/1471-2229-4-16

**Published:** 2004-09-17

**Authors:** Luigi Tedone, Robert D Hancock, Salvatore Alberino, Sophie Haupt, Roberto Viola

**Affiliations:** 1 Unit of Plant Biochemistry, Scottish Crop Research Institute, Invergowrie, Dundee DD2 5DA, UK; 2 Dipartimento di Scienze delle Produzioni Vegetali, Universita degli Studi di Bari, Italy; 3 University of Naples "Federico II", Department of Soil, Plant and Environmental Sciences, Via Universita' 100 – 80055 Portici, Italy

## Abstract

**Background:**

Following on from recent advances in plant AsA biosynthesis there is increasing interest in elucidating the factors contributing to the L-ascorbic acid (AsA) content of edible crops. One main objective is to establish whether in sink organs such as fruits and tubers, AsA is synthesised *in situ *from imported photoassimilates or synthesised in source tissues and translocated via the phloem. In the current work we test the hypothesis that long-distance transport is involved in AsA accumulation within the potato tuber, the most significant source of AsA in the European diet.

**Results:**

Using the EDTA exudation technique we confirm the presence of AsA in the phloem of potato plants and demonstrate a correlation between changes in the AsA content of source leaves and that of phloem exudates. Comparison of carboxyflourescein and AgNO_3 _staining is suggestive of symplastic unloading of AsA in developing tubers. This hypothesis was further supported by the changes in AsA distribution during tuber development which closely resembled those of imported photoassimilates. Manipulation of leaf AsA content by supply of precursors to source leaves resulted in increased AsA content of developing tubers.

**Conclusion:**

Our data provide strong support to the hypothesis that long-distance transport of AsA occurs in potato. We also show that phloem AsA content and AsA accumulation in sink organs can be directly increased via manipulation of AsA content in the foliage. We are now attempting to establish the quantitative contribution of imported AsA to overall AsA accumulation in developing potato tubers via transgenic approaches.

## Background

L-Ascorbic acid (AsA), the reduced form of vitamin C, is an essential antioxidant for many biological systems and must be obtained via the diet by humans, primates and a few other animals which are unable to synthesise AsA endogenously [1]. The main dietary source of AsA for all these organisms are plants and insufficient intake of this micronutrient results in the onset of a debilitating disease (scurvy) and eventually death. In spite of its obvious relevance for humankind, our understanding of how plants synthesise AsA is still rudimentary. It was only in 1998 that an evidence-backed AsA biosynthetic pathway in plants was put forward [2]. The original proposal was supported by the characterisation of *Arabidopsis thaliana *AsA-deficient mutants (*vtc1*) which were defective in the activity of a pathway enzyme [3]. Since then, other AsA-deficient *A. thaliana *mutants have been identified which do not seem to be affected in any of the known pathway genes [4,5]. Additionally, up-regulation of AsA accumulation in plants has been achieved via ectopic over-expression of biosynthetic genes unrelated to the proposed biosynthetic pathway [6-9]. This has led to proposals of additional steps, branches or alternative routes to the original pathway [7,9,10].

Many of the recent advances in plant AsA biosynthesis have been obtained through investigations of model systems such as tobacco or *Arabidopsis thaliana *[2,3,9,11]. On the other hand, our understanding of the mechanisms controlling AsA accumulation in the edible parts of crop plants (e.g. fruits and vegetables), which represent the main dietary source of vitamin C [12] remains limited. One problem is that AsA functions outside the chloroplasts are much less understood compared with those associated with photosynthetic metabolism [13]. We have also no biological or taxonomic explanation for the massive variability in AsA contents in sink organs such as fruits which can contain over 3000 mg/100 g FW in the fruits of camu camu (*Mirciaria dubia*) [14] or less than 10 mg/100 gFW as is the case for grapes, apples or plums [15]. Large variability is also found in non-green vegetables [16] whilst dry seeds are completely devoid of AsA [e.g. [17]]. These examples highlight the high degree of tolerance for AsA content in storage organs and suggest the biological feasibility for the development of AsA-rich crop products. To this end, one key question that needs to be answered is whether AsA accumulation in sink organs occurs as a result of biosynthesis *in situ *or import from the foliage. Recently Franceschi and Tarlyn [18] observed long distance movement of ^14^C-AsA from leaves to flowers and root tips in model systems such as *A. thaliana *and *Medicago sativa*. Research in our laboratory demonstrated the occurrence of AsA in the phloem of a number of crop plants and we also observed that the plant phloem was capable of supporting active AsA biosynthesis from a number of precursors [19]. We have previously shown that rates of AsA accumulation are highest soon after organ formation in sink organs such as blackcurrant berries [20] and potato tubers [21] when sink activity is strongly induced [22]. Taken together, these findings indicate that sink-source relationships may play an important role in defining AsA accumulation in sink organs.

In this work we have extended our investigation on AsA accumulation in potato tubers, the main source of vitamin C in the European diet [23]. We present here evidence for the implication of long-distance transport in AsA accumulation in potato tubers. Additionally, we show that artificial increase of AsA content in the foliage results in phloem AsA enrichment and AsA accumulation in tuberising stolons. Our findings have implications for the development of strategies for increasing the nutritional value of crop plants.

## Results

### Detection of AsA in the phloem

**Figure 1:**
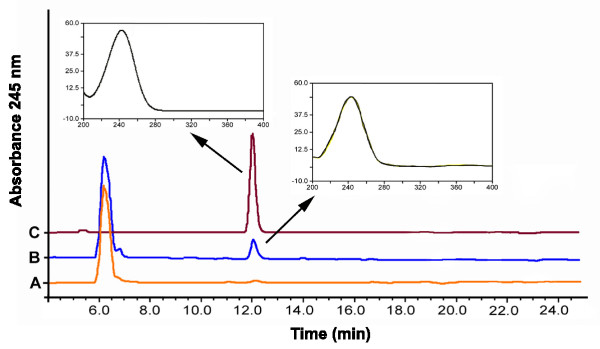
**HPLC traces of potato leaf exudates. **The terminal leaflets of potato leaves were excised from glasshouse plants, petioles were re-cut under water and the freshly cut end transferred to 200 μl 5 mM CaCl_2 _(trace A) or 200 μl 15 mM EDTA (trace B). Samples were transferred to a saturated atmosphere to exude in the dark for 90 min. The exudation medium was brought to 5% MPA, 5 mM TCEP, centrifuged and injected onto a Coregel 64H column. Traces were recorded at 245 nm using a diode array detector and insets show the absorbance spectra of the peaks at 12.2 min. Trace C was authentic AsA (2.27 nmol) which gave a peak height of 42 mAU. The unbound peak at 6.3 min contained MPA.

**Figure 2:**
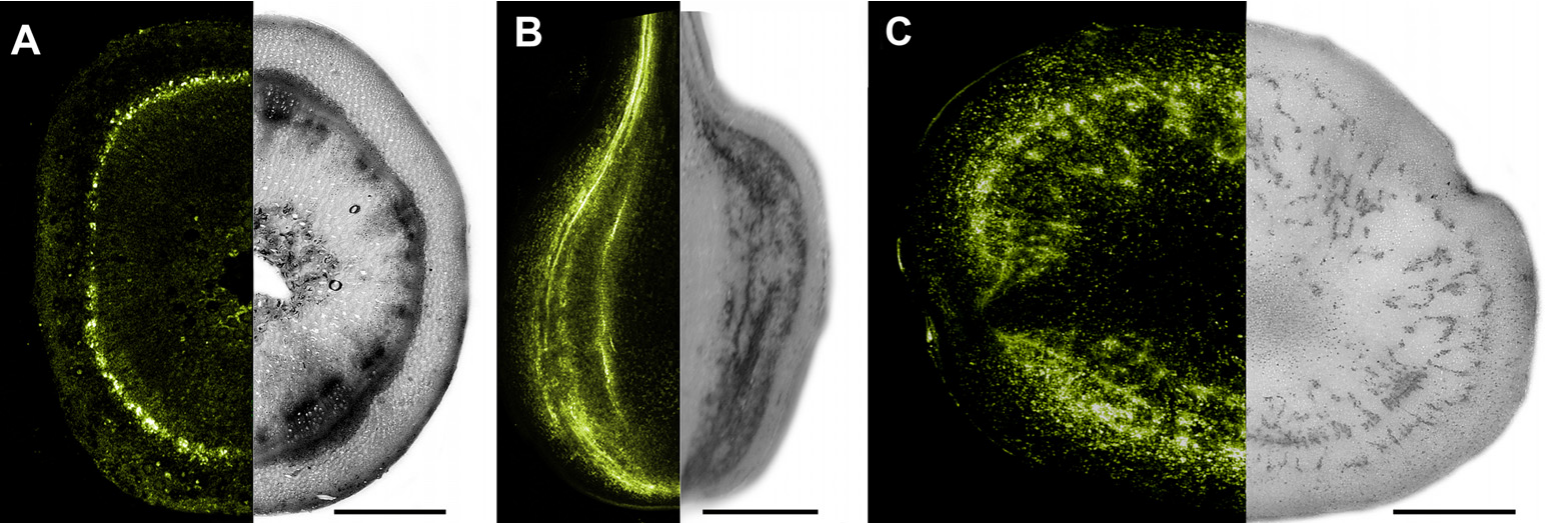
**Comparison of CF and AgNO_3 _staining in potato tissues. **Plants were labelled with aqueous CFDA and left to translocate for 5 h prior to harvesting. Tubers or stems were hand sectioned and sections were either incubated in mineral oil and examined under a confocal microscope for CF fluorescence or stained for AsA using ethanolic AgNO_3_. Confocal images are shown on the left and silver stained sections on the right of each panel. A, stem (bar = 2 mm); B, developing tuber (bar = 3 mm); C, large developing tuber (bar = 5 mm).

Fig. 1 shows HPLC chromatograms of potato source leaf phloem exudates collected from severed petioles in buffer containing either EDTA or CaCl_2_. Total AsA (AsA_t_; L-ascorbic acid + dehydroascorbic acid) appeared as the largest peak of absorbance at 245 nm retained by the column in both cases. When exudates were collected in the presence of CaCl_2 _instead of EDTA, all exudate derived peaks were strongly reduced due to the reduction in exudation caused by callose gelation [24]. In experiments to test the stability of authentic AsA (final concentration 0.1 μM) in the presence of EDTA or CaCl_2 _exudation buffers less than 5% oxidation was observed in either case over 90 min (data not shown). AsA localisation to the vascular tissue was confirmed histochemically in sections of potato stems and tubers incubated with ethanolic AgNO_3 _at 3°C (Fig. 2). Intense deposition of metallic silver was observed in the vasculature of stems. In tubers metallic silver deposits appeared as short strands or well defined spots, sometimes in the perimedullary zone and also in the cortex. Although AgNO_3 _staining and CFDA treatments could not be carried out on the same section due to interference between the treatments, a clear similarity was observed in the pattern of metallic silver deposits and the distribution of fluorescence in stem and tuber sections. No AgNO_3 _staining was observed in control sections pre-incubated with 1% CuSO_4 _for 18 h in order to oxidise AsA (data not shown) [25].

### AsA distribution during tuber development

**Figure 3:**
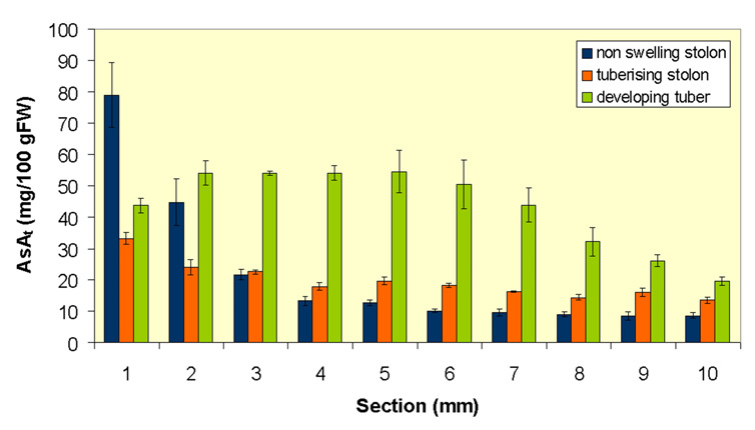
**Distribution of AsA_t _along the axes of stolons and developing tubers. **Non-swelling stolons, tuberising stolons or developing tubers were excised from glasshouse grown plants and sliced into 1 mm transverse sections which were quickly weighed and immediately frozen in liquid nitrogen. Samples were ground to a powder, extracted in 5% MPA, 5 mM TCEP (9:1 v/w) and AsA_t _in the extract supernatant estimated by HPLC. Values are represented as mean ± SE, n = 3.

Fig. 3 shows AsA_t _distribution along the axis of stolons and developing tubers. In non-swelling stolons the AsA_t _content was maximal in the apical section with a sharp basipetal decline such that the more basal sections contained approximately 10% of the AsA_t _content found in the apex. Tuberising stolons showed a 50% reduction in the AsA_t _content of the apical section. This was accompanied by increases in the subapical 3–10 mm sections. In developing tubers there was a very substantial increase in the AsA_t _content in the sub-apical region, corresponding to the swelling area.

### AsA transport

**Figure 4:**
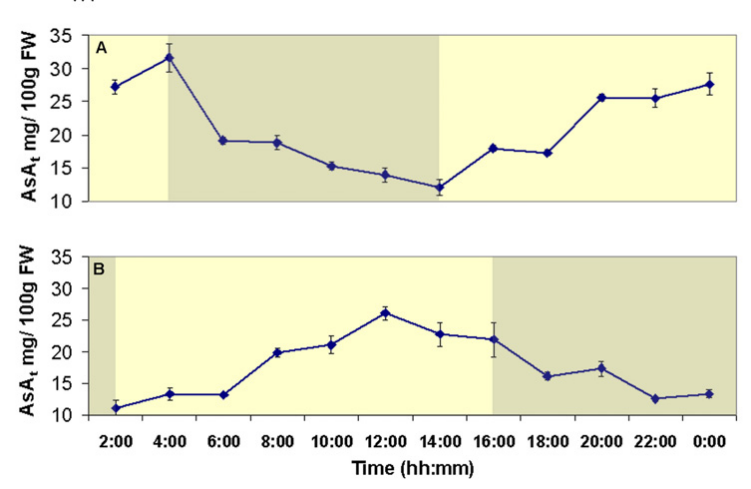
**Changes in AsA_t _content of potato leaves as a function of light. **Glasshouse grown plants were transferred to off-phased controlled environment chambers (panels A and B) 14 days prior to the start of the experiment. Environment chambers were on a 10 h dark – 14 h light cycle as indicated by the brown and yellow panels respectively. At the times shown, source leaves (leaves on the lower four nodes of each stem) were removed from each of three plants, ground in liquid nitrogen and extracted in 5% MPA, 5 mM TCEP (9:1 v/w) prior to quantification by HPLC. Values are represented as mean ± SE, n = 3.

Figure 4 shows the changes in AsA_t _content of source leaves of potato plants acclimatised for 14 days in cabinets with artificial diurnal dark/light cycles. The cabinets were off-phased so that plants at different stages in their lighting regime were available at any given time. The AsA_t _content of source leaves was monitored for 24 h in plants sampled simultaneously from the two cabinets. At the end of the dark phase, leaf AsA_t _content was approximately 12 mg/100 gFW and it progressively increased following artificial sunrise. After 10 h of light, the leaf AsA_t _content increased to approximately 30 mg/100 gFW after which it leveled off. Following artificial sunset the AsA_t _level gradually fell back to approximately 12 mg/100 gFW within 6–10 h.

Table 1 shows the AsA_t _content of source leaves and tuberising stolons collected at 12.00 h from the light-phase or dark-phase plants (after collection of phloem exudate for 90 min in a prehumidified atmosphere in leaves). In the table are also reported the chromatographic AsA_t _peak areas from phloem exudates collected from source leaves and tuberising stolons of the same plants. The exudate data are reported as peak areas as the exact volume of exudate collected could not be established. Whilst the leaves from light-phase plants contained over twice the AsA_t _level of leaves from dark-phase plants, no significant difference was found in the AsA_t _content of tuberising stolons from the two sets of plants. The AsA_t _peak area in chromatograms of light-phase leaf exudates was 1.8-fold larger than that obtained from dark-phase leaves. In tuberising stolons the increase in exudate peak area was more pronounced (4.6-fold).

**Figure 5:**
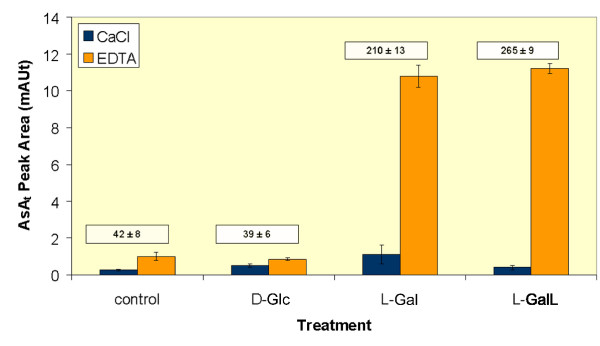
**AsA_t _content of petiole exudates after treatment of source leaves with AsA precursors. **Terminal leaflets of glasshouse grown potato plants were supplied with 500 μl of the substrate indicated (25 mM) via a 'flap' whilst still attached to the plant. After 24 h incubation, leaflets were removed for AsA_t _determination in tissues and collection of exudates (90 min). Bars represent the mean exudate AsA_t _peak area ± SE (n = 4) and values in boxes represent the mean tissue AsA_t _content (mg/100 gFW) ± SE (n = 4). mAUt = milli-absorbance units (λ245 nm) × time

The correlation between leaf AsA_t _content and the AsA_t _levels of phloem exudates was also investigated in glasshouse-grown plants following the supply of a range of AsA biosynthetic precursors using the flap technique [18]. Figure 5 shows the chromatographic AsA_t _peak area of exudates from leaves pre-treated for 24 h with 20 mM MES pH 5.5, 2 mM CaCl_2 _alone (control) or containing precursors at a final concentration of 25 mM. Incubation with D-glucose (D-Glc) resulted in a slight (10%) reduction in leaf AsA_t _content compared with the control but no significant change in AsA_t _was detected in the exudates. By contrast, supply of L-galactose (L-Gal) or L-galactono-1,4-lactone (L-GalL) increased the AsA_t _content of leaves (4.9 and 6.2-fold respectively) and, more substantially, of exudates (10.8 and 11.2-fold respectively). With all treatments, the replacement of EDTA with CaCl_2 _in collection wells resulted in a significant reduction of AsA_t _peak area in the exudate chromatograms.

**Figure 6:**
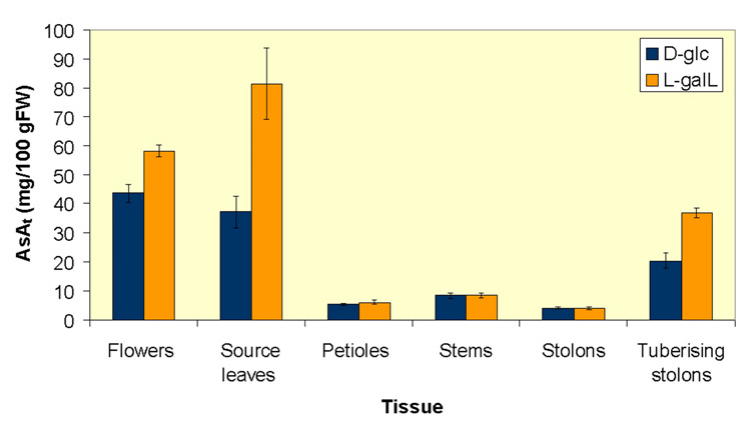
**Effect of precursor supply to source leaves on AsA_t _content in sink tissues. **Terminal leaflets of all leaves of the lower four nodes of each stem of four potato plants were incubated with 500 μl 20 mM MES pH 5.5, 2 mM CaCl_2 _alone (control) or containing 25 mM L-GalL for 24 h. At the end of incubation the indicated tissues were removed from plants, snap frozen in liquid nitrogen and lyophilised. After lyophilisation, tissues were powdered and extracted in 5% MPA, 5 mM TCEP (19:1 v/w) prior to estimation of the AsA content in each tissue by HPLC. Values are presented as means ± SE, n = 4.

An artificial increase of AsA_t _content in the totality of source foliage of whole plants was obtained through the supply of the direct AsA precursor L-GalL via the flap technique for 24 h to all terminal leaflets of the four lowermost nodes. At the end of the incubation period the AsA_t _content of source leaves was 2.2-fold higher than in control plants (Fig. 6). AsA_t _content also significantly increased in sink organs such as flowers (33%) and, more substantially in developing tubers (80%) compared with the control. No changes in AsA_t _levels were observed in petioles, stems or non-tuberising stolons.

## Discussion

### AsA in the potato phloem

We have previously shown that AsA represented the largest peak of absorption at 245 nm retained by the column when phloem sap of potato source leaves obtained by aphid stylectomy was analysed by HPLC [19]. In the present study we obtained very similar results from phloem samples collected from different organs of the potato plant by a different approach i.e. the EDTA exudation technique [24]. Localisation of AsA to the vascular tissue of stems and developing tubers was further demonstrated histochemically exploiting the specific interaction between AsA and AgNO_3 _at low temperature with ensuing formation of metallic silver deposits [25]. In particular, the distribution of metallic silver in developing tubers generated a pattern reflecting the intense phloem anastomosis typical of these storage organs [26] and resembling the phloem network involved in the unloading of labelled assimilates in plants supplied with ^14^CO_2 _[22]. This was further evidenced by the close similarity between the silver nitrate staining and distribution of fluorescence in tubers following supply of CFDA to source leaves. We have previously demonstrated that the distribution of CF in developing potato tubers can be used to identify symplastic phloem unloading in these organs [22], thus the similar distribution of CF and metallic silver deposits in developing tubers implies that transfer of phloem AsA to storage parenchyma cells occurs with the mass flow of assimilates. This hypothesis is further supported by the changes in AsA_t _distribution along the stolon axis following tuber induction which closely resemble the changes in sucrose content and radiolabelled assimilate distribution observed in plants labelled with ^14^CO_2 _[22]. The decline of AsA_t _in the apices of tuberising stolons accompanied by its accumulation in the subapical region may thus reflect the induction of symplastic phloem unloading in this region resulting in a distal migration of sink activity from the apex. However, this pattern also correlates with changes in the mitotic index along the axis as a result of tuber induction with cessation of cell division in the apical region of the stolon [27] and its activation in secondary meristems within the swelling region [28]. This may be relevant in view of the purported role of AsA in cell division [29].

### Long-distance AsA transport in potato

In preliminary experiments, we observed diurnal changes in the AsA_t _content of mature source leaves of potato. Circadian or diurnal oscillations of AsA content in photosynthetic tissues are known [e.g. [30]] as well as light-induction of AsA accumulation in leaves [e.g. [31]] and sink organs. Light-induced expression of specific AsA biosynthetic enzymes has been observed [e.g. [30]] as well as a general increase in the AsA biosynthetic flux [11]. By growing plants in cabinets with out-of-phase light/dark regimes we were able to simultaneously sample plants in the dark or light phase when maximal differences in foliage AsA_t _content occurred. Leaf AsA_t _content showed diurnal changes with the maximal levels (observed during the light-phase) 2-fold higher than the lowest levels observed in the dark. Phloem exudates obtained from source and sink organs of light phase plants always showed significantly higher AsA_t _levels than exudates obtained from dark phase plants. Indeed, the AsA_t _content of exudates from tuberising stolons of light phase plants was over 4-fold higher that of dark-phase plants. Our findings indicate that changes in source leaf AsA biosynthesis rapidly impact on the phloem AsA_t _content resulting in transport of *de novo *synthesised AsA directly to developing sinks. This was also confirmed in experiments where exogenous AsA precursors such as L-GalL or L-Gal were supplied to source leaves via the flap technique, a treatment which resulted in substantial AsA_t _enrichment in phloem exudates. We were also able to more than double the AsA_t _content of the source foliage of whole plants by "bulk" supply of exogenous L-GalL to the majority of source leaves for 24 h. This resulted in significant AsA_t _increases in sink organs such as flowers and developing tubers.

To our knowledge this is the first demonstration that AsA accumulation in the foliage results in AsA increases in storage organs and it may explain the positive effect of light irradiation on AsA content of fruits and vegetables [32]. The rapid changes in phloem AsA concentration which reflected changes in mesophyll AsA content indicate that AsA storage in the foliage does not occur, unlike the case of assimilated carbohydrates. It is tempting to speculate that AsA produced within mesophyll cells is directly taken up by SE/CC complex for translocation, as also deduced from the results reported by Franceschi and Tarlyn [18]. The kinetics of AsA transport have been studied across a number of plant membranes such as the plasmlemma, the chloroplast membrane, the thylakoid membrane and the tonoplast [33]. However, to date no transporter has been identified although at least 12 genes encoding putative nucleobase/ascorbate transporters (NATs) have been identified in the *Arabidopsis *genome. NATs belong to a superfamily of integral membrane transporters, which move purines, pyrimidines or ascorbic acid across biological membranes and which also comprises mammalian transporters specific for ascorbate [34]. Functional characterisation of these transporters is in progress [35]. As the plant phloem contains the complete enzymic complement for AsA biosynthesis [19], we have already speculated that the indirect transfer of AsA from the mesophyll to the phloem could involve the transport of the non-charged intermediate L-Gal across membranes. We show here that exogenous supply of L-Gal or L-GalL to source leaves leads to AsA enrichment of phloem exudates and AsA accumulation in tuberising stolons and developing tubers. Furthermore, we have observed uptake of L-[1-^14^C]Gal by source phloem of potato (data not shown), as already demonstrated in *N. benthamiana *[19].

## Conclusions

The evidence we provide here for long-distance transport of AsA in potato plants, corroborates earlier findings in *A. thaliana *and *M. sativa *[18] and suggests that source-sink AsA translocation may be a general occurrence in plants. What remains to be established is the relative contribution of phloem-derived AsA (whether synthesised in the mesophyll or in the phloem itself) to overall AsA accumulation in potato tubers. Slices excised from developing potato tubers can synthesise AsA from a variety of substrates (Hancock and Viola, unpublished). Moreover, microtubers obtained *in vitro *from nodal cuttings cultured in the dark with sucrose as the sole carbon source contain similar amounts of AsA as field-grown tubers [21]. These latter findings in particular are difficult to reconcile with the hypothesis of a major role played by long-distance AsA transport in tuber AsA accumulation. The development of transgenic potato plants with tissue and organ-specific down-regulation of AsA biosynthesis will be required to address this issue.

## Methods

### Plant material and growth conditions

Potato plants cv. Desiree were grown in 40 cm pots in unheated glasshouses under natural light in compost. In order to subject plants to artificial light/dark cycles, plants were transferred after 35 days to Sanyo Fitotron 1700 controlled environment cabinets and maintained for a further 14 days on 14 h–10 h light-dark cycles with day and night temperatures of 22°C and 15°C respectively. Light was provided by 60 W incandescent lamps to provide a photon flux of 900 μmol m^2 ^s^-1 ^at the top of the canopy. Relative humidity was maintained at a constant 70% and plants were watered daily. In all cases experiments were performed on tuberising plants after 40–60 days from planting. Throughout the text stolons are defined as non-swelling (uniform diameter along terminal 15 mm) or tuberising (swelling 2–5 mm diameter). Swellings between 5–10 mm diameter are defined as developing tubers.

### Quantification of AsA in plant tissues

Tissue was extracted in a mortar and pestle with ice-cold 5% metaphosphoric acid (MPA) containing 5 mM tris(2-carboxyethyl)phosphine hydrochloride TCEP (9:1 v/w). Samples were then held on ice for 60 min to allow reduction of dehydroascorbic acid to AsA therefore, all data are reported as total AsA pool (AsA_t_) i.e. reduced L-ascorbic acid + dehydroascorbic acid. Samples were then centrifuged at 16000 g for 5 min at 1°C and AsA_t _in the supernatant quantified by HPLC according to the method of Hancock et al [36]. Briefly, 20 μl of sample supernatant were injected onto a 300 × 7.8 mm ID Coregel 64H ion exclusion column (Interaction Chromatography, San Jose, CA, USA) with a 4 × 3 mm ID carbo-H^+ ^guard cartridge (Phenomenex, Macclesfield, UK) maintained at 50°C. Mobile phase was 8 mM H_2_SO_4 _at 0.6 ml min^-1 ^and AsA_t _was detected at 245 nm using a Gynkotech UVD 340S diode array detector (Dionex, Camberley, UK).

### Detection of AsA in the phloem

Phloem exudates were collected from the petiole of source leaves or tuberising stolons using an adaptation of the method developed by King and Zeevart [24]. Following excision of the organs, a portion of the petiole (5 mm) or stolon (10 mm) was removed under water, the sample was rinsed and the cut end transferred to a 0.6 ml reaction tube containing 200 μl 15 mM EDTA pH 7.5. In the case of petioles, samples were transferred to a pre-humidified atmosphere at 20°C and exudate collected for 90 min in the dark. In the case of stolons, exudates were collected from the cut end which remained attached to the plant and moist paper was wrapped around the top of the reaction tube to minimise evaporation. Control samples were run in parallel in which petioles or stolons were incubated in 5 mM CaCl_2 _pH 7.5 to induce callose gellation and reduce exudation [24]. At the end of the incubation, MPA and TCEP were added to the samples to a final concentration of 5% and 5 mM respectively. Following centrifugation (16000 g, 1°C, 5 min), AsA_t _concentration was determined by HPLC as described above. Histochemical localization of AsA in tubers using the AgNO_3 _method was carried out as previously described [19]. Briefly, tubers were hand sliced to form approximately 2 mm sections, washed in distilled water and fixed and stained in 5% (w/v) AgNO_3 _dissolved in 66% (v/v) aqueous ethanol containing 5% (v/v) glacial acetic acid at 3°C in the dark for up to 24 h. The reaction was stopped by washing the tissue twice for 15 min in ethanolic ammonium hydroxide (95% (v/v) 70% ethanol, 5% (v/v) NH_4_OH ACS reagent, Sigma-Aldrich, Dorset, UK) [25]. Finally the tissue was transferred to 70% (v/v) ethanol and stored at 3°C prior to photography.

### Transport of carboxyfluorescein in potato plants

Phloem transport through potato stems into developing tubers was visualised using the fluorescent transportable molecule carboxyflourescein (CF) as previously described [22]. Plants were labelled with 20 ml of an aqueous carboxyflourescein diacetate (CFDA) solution (1 mg ml^-1^) introduced via open stomata on the abaxial leaf surface using a plastic syringe. The acetylated compound is able to diffuse across cell membranes unlike its deacetylated derivative CF which is produced in vivo by endogenous esterases and is used as a marker for phloem strands and symplastic unloading from the phloem [37]. Plants were left to translocate CF for 5 h prior to hand sectioning (2 mm) and examination of stem and sink tissues for fluorescence using a MRC2000 confocal microscope (Bio-Rad, Hemel Hemstead, UK).

### Supply of precursors to leaves

Leaf AsA_t _levels were manipulated using an adaptation of the 'flap' technique [18]. An incision (15 mm) was made either side of the midrib of terminal leaflets and the 'flap' formed was placed into a 0.6 ml eppendorf tube containing 500 μl of 20 mM MES pH 5.5, 2 mM CaCl_2 _alone or with the addition of various intermediates at a final concentration of 25 mM for 24 h. At the end of the incubation period, leaflets were ground in liquid nitrogen and extracted in 5% MPA containing 5 mM TCEP (9:1 v/w) and the AsA_t _content measured by HPLC. For measurement of phloem exudates, treated leaflets were excised under water and placed in eppendorf tubes for collection of phloem exudates as described previously. In some experiments the terminal leaflets of the four lower nodes on all stems (between 8 and 10 per plant) were simultaneously supplied with 500 μl 20 mM MES pH 5.5, 2 mM CaCl_2 _alone or containing 25 mM L-GalL for 24 h. Four independent plants were used for each treatment. At the end of the incubation individual plants were separated into flowers, source leaves, leaf petioles, plant stems, non-tuberising stolons (terminal 15 mm), or tuberising stolons (swelling portion). Tissues were immediately frozen in liquid nitrogen and lyophilised. Lyophilised tissue was ground to a powder and 3 × 1 g fractions of each tissue were extracted in 5% MPA, 5 mM TCEP (19:1 v/w) and the AsA_t _content of each extract analysed by HPLC to give an average value for each tissue. No developing tubers larger than 5 mm diameter were present in the plants.

## Authors' contributions

LT undertook most of the physiological and biochemical experiments. RDH participated in the design and coordination of the study, the biochemical experimentation and the writing of the manuscript. SA participated in the physiological, biochemical and histochemical experiments. SH undertook the CFDA labeling and silver staining of potato plants and participated in production of the figures. RV conceived the study, participated in its design and coordination and drafted the manuscript.

## Figures and Tables

**Table 1:** Effect of light on AsA_t _content in source leaves, tuberising stolons and exudates

Tissue	Cycle	**AsA_t _in tissue (mg/100 gFW)**	**AsA_t _in exudate (peak area mAUt)**
Leaf	Light	42.5 ± 3.1	1.1 ± 0.1
	Dark	19.1 ± 3.0	0.6 ± 0.05
Tuberising stolon	Light	4.1 ± 0.3	7.9 ± 0.8
	Dark	4.5 ± 0.4	1.7 ± 0.9

Glasshouse grown plants were transferred to off-phased controlled environment chambers 14 days prior to the start of the experiment. Environment chambers were on a 10 h dark – 14 h light cycle (see Fig. 4). At 12:00 h source leaves and tuberising stolons were removed from 3 plants and a sub-sample used for tissue AsA_t _quantification. For the determination of AsA_t _in leaf phloem exudates, petioles were re-cut under water and placed into EDTA or CaCl_2 _exudation buffer for 90 min in a prehumidified chamber in the dark. For determination of stolon phloem exudates the cut end of the stolon attached to the plant was re-cut under water and placed in the appropriate exudation solution. Values are represented as mean ± SE, n = 6. mAUt = milli absorbance units (λ245 nm) × time.
